# Rethinking heart failure clinical trials: the heart failure collaboratory

**DOI:** 10.3389/fcvm.2024.1350569

**Published:** 2024-01-24

**Authors:** Mutaz Alkalbani, Mitchell A. Psotka

**Affiliations:** Department of Cardiology, Inova Schar Heart and Vascular, Falls Church, VA, United States

**Keywords:** heart failure collaboratory, heart failure, clinical trials, academic research consortium, HFrEF—heart failure with reduced ejection fraction, HFpEF—heart failure with preserved ejection fraction

## Abstract

The Heart Failure Collaboratory (HFC) is a consortium of stakeholders in the heart failure (HF) community that aims to improve the infrastructure of clinical research to promote development of novel therapies for patients. Since its launch in 2018, HFC has implemented several solutions to tackle obstacles in HF clinical research including training programs to increase the number of clinicians skilled in conducting clinical trials, novel study designs, and advocacy for a diverse and inclusive HF research ecosystem. We highlight some of the HFC successes since its establishment.

## Introduction

The incidence of heart failure (HF) in the United States is on the rise. The American Heart Association estimates about 6.2 million American adults were living with HF between 2013 and 2016 (1). In addition to the increasing mortality and morbidity in HF, the burden of the disease on the economy is significant, including the direct cost to healthcare systems and indirect cost on society related to missed days of work. In 2012 the American Heart Association report estimated the cost of HF on the United States exceeded $30 billion (2). Globally, data on the incidence of HF is limited or even missing. However, the prevalence of HF globally is on the rise due to advancements in diagnostic tools which is also associated with a significant increase in the cost of care (3). Despite being a national and international public health issue, the development of therapies and devices targeted at the prevention and treatment of HF are straggling. This is in part due to the flaws in the infrastructure of HF clinical research in the United States (4).

HF has become difficult to study due to several patient and disease factors including the complexity of the disease and the increase in the expenses associated with conducting research studies. HF is a composite of several diseases and phenotypes that were previously seen as one single entity. Investigational studies have demonstrated the heterogeneity of the disease and the variable effectiveness of the current guideline-directed therapy.Clinical trials in HF require the enrollment of large sample populations with long follow up periods to collect sufficient numbers of events to demonstrate a treatment effect. The conduct of safe, effective, and generalizable clinical trials is challenging due to several other factors. Patient enrollment may be difficult in part due to hesitancy to receive experimental therapies, the long duration of follow up is a deterrent, and clinical trials are complex for patients and clinicians to understand. In addition, highly specific inclusion, and exclusion criteria limit enrollment, which eventually leads to delayed testing of new therapies and increases the cost of running the clinical trial. For example, several epidemiological studies have demonstrated the increase in the incidence of heart failure with preserved ejection fraction (HFpEF) globally (3). Despite the increase in the number of HFpEF trials, most of these trials are limited in size and lack the capacity to generalize the outcomes on HFpEF patients population (5). The inclusion criteria in some of these trials limited the number of HFpEF patients eligible for enrollment (6).

There is an incremental loss in the workforce capable of designing and implementing clinical trials to generate evidence. Fewer clinicians participate in clinical trials as investigators in part due to lack of incentives, resources, and protected time. The high complexity of clinical trials adds extra burden on clinicians who attempt to integrate clinical research into daily practice. While the number of patients with HF is on the rise, there is a decline in the number of physicians trained in management of HF, leading to limited physician-patient interaction time, which also limits the time during which clinicians can discuss clinical trials with patients. Furthermore, stakeholders in clinical trials including health systems, regulatory boards, industry, and third-party sponsors are not in alignment when it comes to defining protocols, end points, and adverse outcomes (7). Additionally, the cost of developing novel therapies is on the rise. It is estimated that it costs industry $2.5 billion to develop a new therapy due in part to the cost required to run phase I, II, and III trials to demonstrate the effectiveness and safety of the therapy. This is linked with the increased cost of enrolling each participant, which has increased four-fold over the past 20 years.

## The heart failure collaboratory

**Figure 1:**
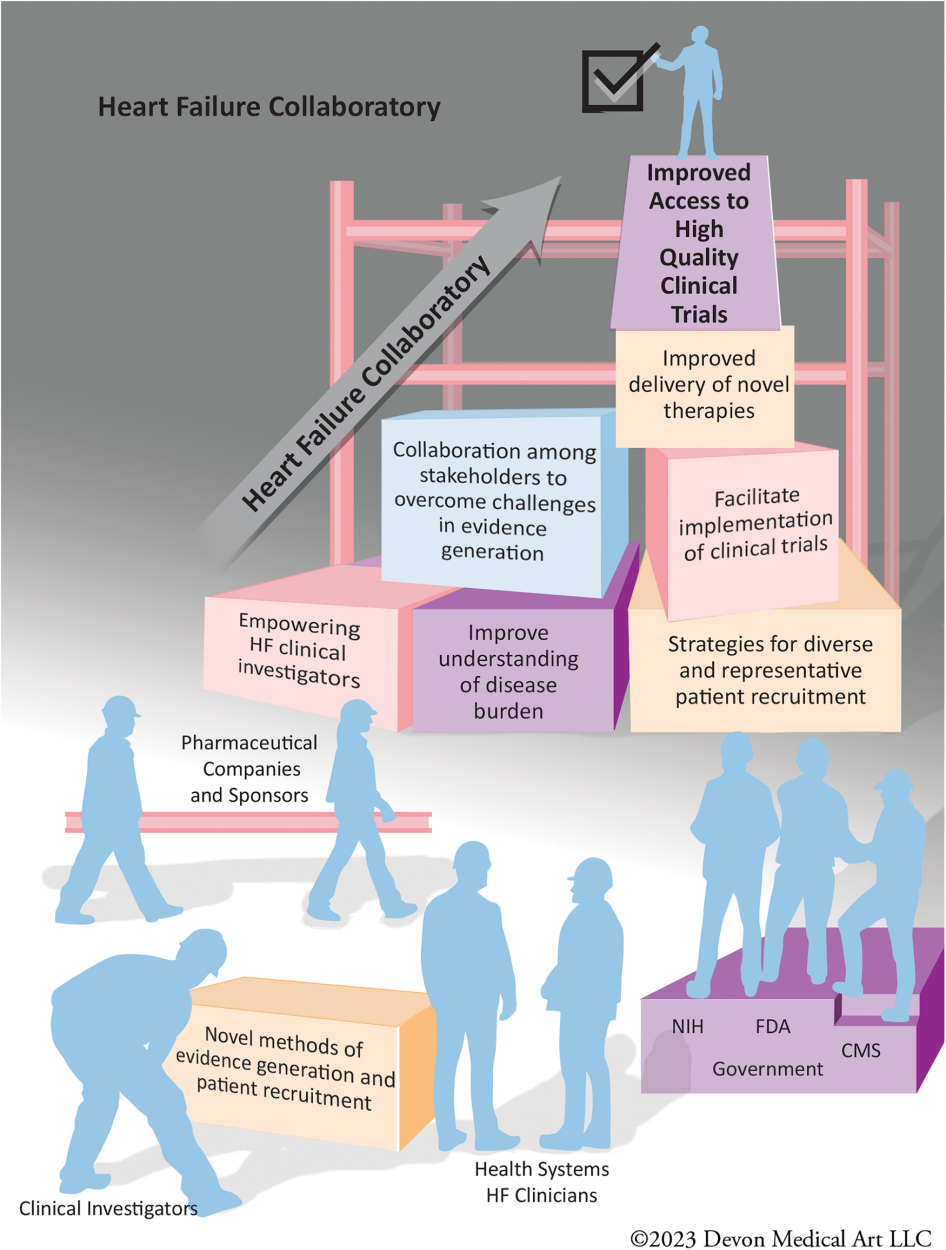
The stakeholders of the heart failure collaboratory working together to improve the infrastructure of heart failure clinical trials.

In order to address these challenges in the HF clinical research environment, the HF Collaboratory (HFC) was launched in 2018. The HFC is a consortium of stakeholders committed to improving the ecosystem of HF clinical trials, which originated from a Think Tank meeting held on March 31, 2017 (4). HFC is a public-private partnership with a collaboration of stakeholders in HF clinical trials including health systems, clinical investigators, pharmaceutical and device companies, society representatives, and governmental agencies including United States Food and Drug Administration (FDA), National Institutes of Health (NIH), and Centers for Medicare and Medicaid Services (CMS). HFC addresses the deficiencies within the infrastructure of HF clinical science, and through learnings and publications described herein has become a model for improving evidence generation for multiple disease processes (Figure 1).

**Figure 2:**
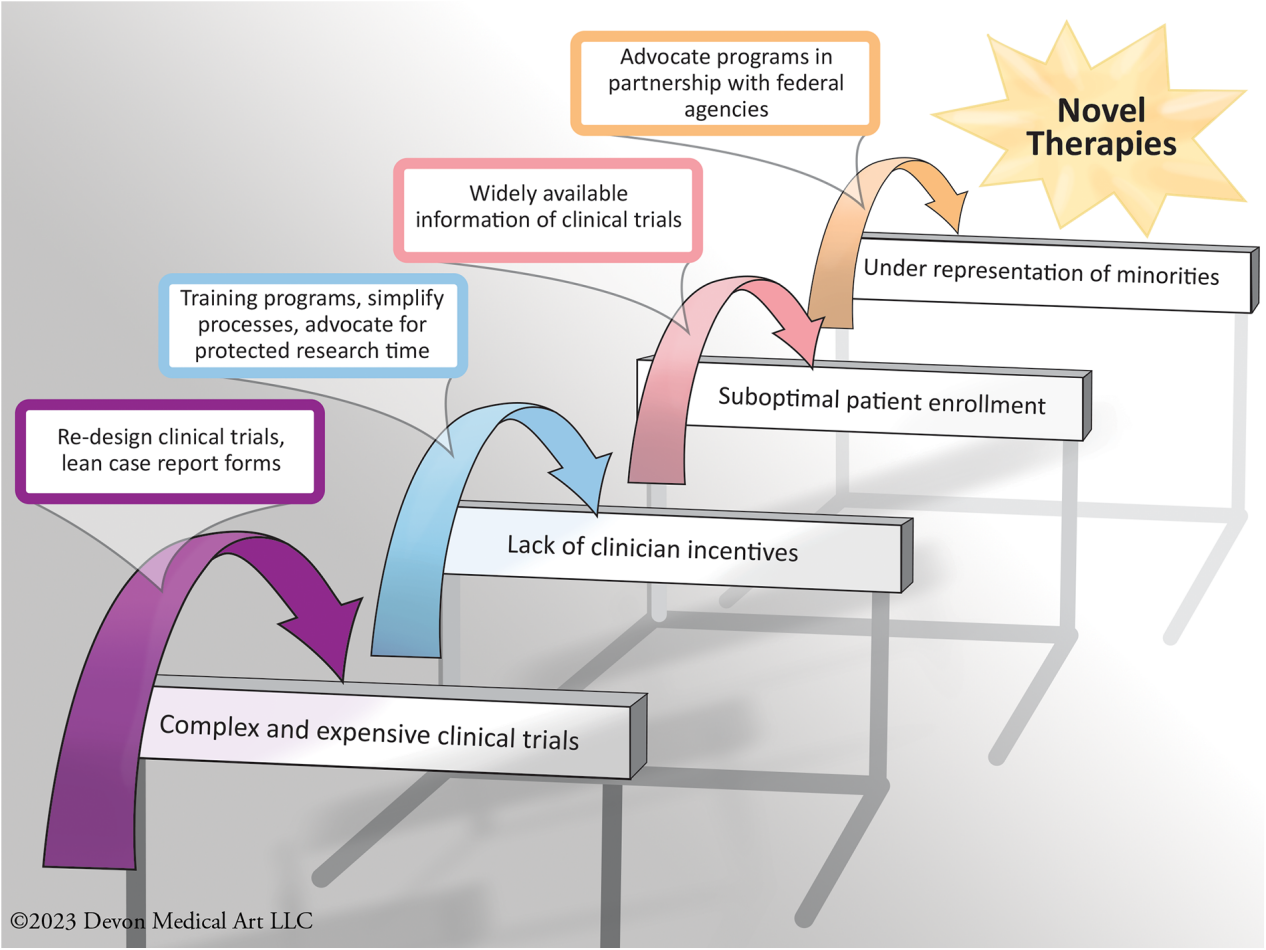
Some examples of the challenges commonly faced in the infrastructure of heart failure clinical trials, with some of the solutions implemented by the heart failure collaboratory.

The HFC aims to improve patient care in the HF ecosystem by augmenting clinical research efficiency and rigor, develop an infrastructure for clinical research, enhance the design of clinical trials, and facilitate the efforts of all stakeholders to improve medical care in HF to facilitate evidence generation for novel HF therapies. HFC is headquartered and administrated at Inova Schar Heart and Vascular (ISHV). The HFC activities enhance enrollment of patients, train future generations of clinical trialists and investigators, and enrich communication across all stakeholders by bringing them together to achieve their collective goals (Figure 2).

### Lessons learned from other disease states

Similar to HF, the incidence of cancer is on the rise. The Center for Disease Control and Prevention estimates that over 1.6 million Americans were diagnosed with cancer in 2020 with over 600,000 cancer deaths (8). Due to the fast-paced development of new therapies to detect, prevent and treat cancer, there was a critical need for systematic reforms to coordinate the efforts of clinical trials in cancer therapies. In 2010, the National Comprehensive Cancer Network and the oncology community developed a road map to improve clinical trials in oncology by unifying the national efforts in cancer research. This included the standardization of clinical trials and generating funds and supportive resources to facilitate the implementation of new therapies in clinical practice (9).

Cystic fibrosis therapeutic development also offers a model for enhanced therapeutic development by disease-state stakeholders. The Cystic Fibrosis Foundation launched the CF Therapeutics Development Network in 1998 that acts as a facilitator between pharmaceutical companies, health systems, and patients to deliver novel therapies in a timely fashion. This network oversees the funding and investments of revenues from cystic fibrosis therapies and focuses on the empowerment of patients (10). Successes in the oncology and cystic fibrosis environments of collaborative multi-stakeholder community organizations led directly to the creation of the HFC.

## Training programs

Clinical investigators and researchers are the driving force behind clinical trial patient enrollments. Investigators understand the challenges in the management of HF, utilize the therapies available on the market, and select the appropriate therapy for the right patient. The longitudinal patient-physician relationship in HF is an important aspect to prevent HF, identify it when present, and understand its progression. However, fewer HF clinicians enroll in clinical trials than previously. As the population ages the prevalence of HF continues to rise, exacerbating demand and supply mismatch in HF care. Additionally, the number of trainees in advanced HF has plateaued and a significant number of training positions have remained unfilled (11). This insufficiency combined with the heavy patient volume has decreased access to care, increased the burden of clinical work, and reduced the time allocated for clinical research. The complexity of clinical trials and lack of incentives also contribute to the diversion away from clinical research. Academicians within the HF community have done an astonishing work to advance care in HF, however, far fewer clinicians continue to join this specialized community (12). In order to tackle this problem, the HFC designed and implemented training programs to empower and enrich the current and future generation of clinicians, aiming to enhance the workforce behind designing, implementing, and supervising clinical research in HF.

### Clinical research internship program

The HFC launched the Clinical Research Internship Program targeting young scholars and aspiring medical students to prepare the future generation of academic clinicians and research enthusiasts. The program provides interns with the opportunity to gain the experience in clinical site-based research, with a focus on HF and cardiovascular diseases. The program hopes to incentivize young scholars to change the research culture and promote evidence generation. At the same time, interns benefit from boosting their academic portfolio and connecting with leaders in the field. Interns participate in HFC working group meetings, partner in research generation, help maintain the HFC social media platforms to broadcast HFC activities, and collaborate in the production of HFC end-products and manuscripts.

One HFC aim addressed by past interns is investigating novel methods in the design and analysis of clinical trials. In 2020 Perego et al. published a paper under the supervision of the HFC leadership on the utility of the restricted mean survival time analysis (RMST) in HF clinical trials (13). RMST as defined by the average event-free time up until a milestone time point, is an alternative to Cox proportional hazards modeling that is commonly used in the analysis of HF clinical trials (14). RMST was calculated from the published time-to-event data from landmark clinical trials in HF using Kaplan-Meier survival curves. RMST differences were estimated and compared with proportional hazard models. Finally, Weibull estimations were applied to extrapolate the trials’ data for 5 years of treatment time. In addition, the RMST is also patient-centered, in that it provides easily interpretable assessments of benefit. For instance, using Weibull estimation treatment with dapagliflozin in the clinical trial “Dapagliflozin in Patients with Heart Failure and Reduced Ejection Fraction” was associated with 1.8 added months of life of patients compared to placebo. Alternatively, from the effect of spironolactone on morbidity and mortality in patients with severe heart failure clinical trial (RALES) was associated with 6.0 added months of life compared to placebo (15, 16). By applying this approach in the designs of clinical trials, patients can be provided with intuitive estimates of HF therapies without prohibitive statistical assumptions.

### Data and safety monitoring board workshop

A Data and Safety Monitoring Board (DSMB) is an independent committee that monitors patient data during ongoing clinical trials to ensure safe and effective conduct of the study. A DSMB identifies significant risks or benefits of the therapies under investigation during the experimental phase, and in the case of likely harm or overwhelming evidence of benefit, plays a crucial role in modifying or terminating clinical trials. Members of the DSMB should have the expertise and knowledge in clinical trials as well as the disease process and therapy being investigated (17). However, despite the increased use of DSMB in HF clinical trials, the pool of clinicians trained in DSMB activity is limited and does not satisfy the current or likely future research need. In summer 2023, the HFC launched the DSMB Academy Training Workshop, an initiative that aims to train cardiovascular and HF clinicians in the skills and disciplines of DSMB performance, to increase the pool of DSMB-eligible participants for clinical trials of the future.

## Clinical trial design

### Patient enrollment

Patient enrollment in clinical trials remains challenging due to both trial exclusion criteria and patient reluctance to participate. For example, the Danish German Cardiogenic Shock Clinical Trial (DanGer Shock) is a prospective, multicenter, open-label trial to study left ventricular mechanical circulatory support with a percutaneous implantable microaxial pump in patients with acute myocardial infarction complicated by cardiogenic shock, compared to conventional treatment. The trial was initially approved by the Danish National Ethical Committee in November 2012, and aimed to enroll 360 patients. The first patient enrolled in January 2013, but by the end of June 2018 only 100 patients had enrolled. To overcome the slow enrollment, the study had to be re-designed to involve multiple additional German centers (18). The HFC has highlighted some of the underlying factors behind poor enrollment, including the high complexity of clinical trials and poor patient access to information regarding availability of clinical trials (4). The HFC proposed actions to improve patient enrollment including forming partnerships with patient groups to educate patients on the importance of clinical trials and interventions to develop effective therapies for HF, therapies that may benefit themselves and others suffering from the disease. They also developed a media platform to facilitate patient enrollment where patients can find these trials and apply to participate by themselves, similar to clinical trials for cancer therapies (19).

### Diversity and inclusion

The HFC aims to enhance the generalizability of clinical trials by improving the diversity of patients who participate in clinical trials. Despite the government requirement to report age, race, ethnicity, and sex, participants’ diversity in clinical trials remains suboptimal and under representative of the broader patient population.

Tahhan et al. reviewed 118 HF clinical trials and found that only 27% of participants were female and less than 30% were non-white race. The under representation of women, the elderly, and mixed ethnic groups limits the generalizability of clinical trials and their implementation into clinical practice (5). Social and economic backgrounds also play an important role in the development and progression of HF and potentially the response to therapies, and patients from diverse socioeconomic backgrounds should be included (20). The HFC has advocated for further government legislation. This would help ensure that investigators and sponsors take into consideration methodical study designs, limit eligibility requirements, and promote use of electronic resources and diverse media outlets to enrich trials with diverse participants (4). The HFC also released a call to action to improve the enrollment of underrepresented racial and ethnic populations through the diversification of research leadership and stakeholder commitment (21).

### “Lean” case report form

Large-scale data collection may prolong and complicate the clinical trial process, adding cost that may not be needed if some data elements can be streamlined or removed. Sponsors and investors who fund clinical trials are forced to be selective in choosing therapies to be investigated on cost and investment returns rather than potential efficacy and benefits on the patient population. When designing clinical trials, investigators should consider methods to simplify studies to improve the cost effectiveness and reduce the economic and workforce burden required to run trial. The HFC “lean” Case Report Forms (CRF) specifically for the use in HF clinical trials aim to reduce trial burden by limiting extraneous data collection. A CRF is generally a data capture tool used to catalogue information collected for each study participant in the clinical trial (22). CRFs have historically been extensive, requiring the collection of substantial unnecessary data that increases trial burden. The lean CRF was created after the systematic review of CRFs from 8 HF clinical trials. The lean CRF eliminates non-critical elements, which reduced the number of elements from 176 in the original CRF's to 75 elements. This freely available tool aims to standardize data collection in HF clinical trials and to reduce the cost and burden to run the trials (23).

### Redefining endpoints in HF clinical trials

The HFC partnered with the Academic Research Consortium (ARC), a collaboration between experts in clinical trials and academic research organizations which include the Harvard Clinical Research Institute, Cardialysis, the Cardiovascular Research Foundation, and the Duke Clinical Research Institute, to standardize terminology used in clinical trials. This partnership aims to unify and modernize the endpoint definitions for HF clinical research. This partnership developed patient-centered consensus recommendations for functional and symptomatic endpoints in clinical trials to improve the efficiency of HF clinical trials and potentially lower their cost (24). As one example where additional investigation is needed, there is wide heterogeneity in determining actigraphic measurements in HF clinical trials. As a communal starting point, HFC-ARC published guidelines for actigraphy reporting guidelines that include 16 device attributes including device name and model, sampling rate, events marker, wearable location and monitoring duration (24).

### Lessons from the COVID-19 pandemic

The COVID-19 pandemic that started in late 2019 changed the face of healthcare dramatically including the performance and analysis of clinical trials. For example, the GUIDE-HF trial of pulmonary artery pressure monitoring in HF appeared effective during the portion of the trial conducted before the COVID-19 pandemic but appeared to lose its treatment effect due to a reduction in HF events during COVID-19 pandemic. This finding may be related to patient behavioral changes during the pandemic and not directly related to the therapy monitoring device or COVID-19, but it unmasked the role that external forces may have on the clinical trial ecosystem. Participants may have increased at-home exercise, augmented the quality of homemade meals with reduced salt usage, or increased adherence with medical therapies (25). The HFC and ARC conducted serial meetings in 2020 to address urgent challenges of conducting clinical trials during the COVID-19 pandemic. Members released a timely expert consensus recommendation that reviewed options for maintaining best practices to conduct clinical trials during the pandemic to support ongoing clinical trials and strengthen the clinical trial ecosystem (26). This expert consensus discussed options for remote event monitoring, functional and exercise measures, the effect of COVID-19 infection on endpoints, and special statistical considerations that were used by multiple ongoing and initiating clinical trials at the time and facilitated discussions internally and with regulators.

## Conclusion

As the burden of HF remains on the rise, the HF Collaboratory was launched to improve the development of drugs and devices for its treatment. Despite many ongoing obstacles that slow down or impair evidence generation, including the complexity of current clinical trials, slow patient enrollment, lack of trained and incentivized investigators, diminishing workforce to conduct these trials, and heterogenous definitions for endpoints of clinical trials, the HFC continues to work to overcome these barriers. The HFC has created solutions to tackle these problems through programs to train current and future generations of clinical investigators, re-imaged the design of clinical trials to reduce cost, complexity, and improve patient enrollment, and gathered stakeholders together to support the common cause of improving HF patient care. The HFC is a model for further collaborations among the cardiovascular community to improve therapies, reduce mortality, and improve quality of life for patients.
